# A three‐field monoisocentric inverse breast treatment planning technique without half‐beam blocking

**DOI:** 10.1120/jacmp.v16i5.5494

**Published:** 2015-09-08

**Authors:** Tiezhi Zhang, Joshua T. Dilworth, Ovidiu Marina, Peter Chen, Lisa Benedetti, Qiang Liu

**Affiliations:** ^1^ Department of Radiation Oncology Washington University St. Louis MO; ^2^ Department of Radiation Oncology William Beaumont Hospitals Royal Oak MI USA

**Keywords:** treatment planning, monoisocentric technique, breast cancer

## Abstract

The purpose of this study was to introduce a three‐field monoisocentric inverse treatment planning method without half‐beam blocks for breast cancer radiation treatments. Three‐field monoisocentric breast treatment planning with half‐beam blocks limits the tangential field length to 20 cm. A dual‐isocenter approach accommodates patients with larger breasts, but prolongs treatment time and may introduce dose uncertainty at the matching plane due to daily setup variations. We developed a novel monoisocentric, three‐field treatment planning method without half‐beam blocking. The new beam‐matching method utilizes the full field size with a single isocenter. Furthermore, an open/IMRT hybrid inverse optimization method was employed to improve dose uniformity and coverage. Geometric beam matching was achieved by rotating the couch, collimator, and gantry together. Formulae for three‐field geometric matching were derived and implemented in Pinnacle scripts. This monoisocentric technique can be used for patients with larger breast size. The new method has no constraints on the length of tangential fields. Compared with the dual‐isocenter method, it can significantly reduce patient setup time and uncertainties.

PACS number: 87.55.D‐

## I. INTRODUCTION

The role of adjuvant radiation following lumpectomy or mastectomy in select patients is well documented.^(1)^ When treating with photons, the breast or chest wall is treated with tangential fields. For patients with higher risk of nodal metastasis, a third oblique photon field is used to irradiate the upper axillary and supraclavicular nodal regions. Avoiding a geometric gap or overlap of the three fields poses a substantial technical challenge in three‐field treatment planning.^2^, ^3^, ^4^ Institutions use different methods for field matching. Among them, half‐beam blocking is the most common approach in order to match the supraclavicular field and tangential breast fields.^5^, ^6^, ^7^, ^8^ In this approach, the supraclavicular and tangential breast fields are matched at the midplane of the radiation field. This monoisocentric beam‐matching method with half‐beam block does not require a second setup during treatment delivery, which can reduce treatment time and dose uncertainties in the matching plane.^(5)^ However, the maximum tangential field length is limited to half of the full field size, or 20 cm for most linac machines with this method, which is inadequate for some patients.

Conventionally, a dual‐isocenter, beam‐matching approach is used when tangential fields are longer than 20 cm.^(6)^ In this method, the supraclavicular field is still half‐beam blocked, but the tangential fields use another isocenter located inferior to the matching plane. In order to match with the supraclavicular field, the tangential fields need to have a couch angle and collimator angle to ensure that the superior border of the tangential breast fields matches the inferior border of the supraclavicular field. This dual‐isocenter, three‐field arrangement removes the limitation on the length of the breast fields. This type of treatment delivery requires therapists to walk inside the treatment vault to set up the patient again when switching from the supraclavicular field to the breast fields. The three fields are matched on the patient's skin, where a line is drawn. The therapists move the patient to match the light field edges with the line on the skin. Not only does this procedure prolong the treatment time, it also causes uncertainties in the field matching.^3^, ^9^ The dose gradient on the field edge is very high, and a 1–2 mm mismatch may cause significant dose variation in the matching plane.

We developed a monoisocentric three‐field matching technique without the use of half‐beam blocks. This method eliminates the requirement that the supraclavicular field has to be half‐beam blocked so that the isocenter can be located inferior to the matching plane. Compared with approximate beam matching,^(10)^ the new method always produces perfect geometric matching. The tangential breast fields are geometrically matched with the supraclavicular field by rotating the collimator and couch. Similar to the dual‐isocenter approach, the full‐field length can be utilized for the tangential fields. With a single isocenter, the treatment delivery requires only one setup, thereby treatment time is significantly reduced. More importantly, without manual matching using a light field, the new method reduces dose variation in the matching region due to setup uncertainties.

## II. MATERIALS AND METHODS

### A. Three‐field matching geometry

**Figure 1:**
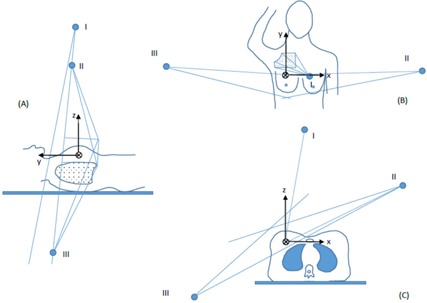
Monoisocentric three‐field matching without half‐beam blocks. The isocenter is located inferior to the matching plane. This method allows monoisocentric setup with perfect geometric field matching for patients with tangential fields longer than 20 cm.

Figure 1 illustrates the new monoisocentric three‐field arrangement. Point I, II, and III are the MV source positions for the supraclavicular, medial, and lateral breast fields, respectively. In this beam arrangement, the isocenter is inferior to the matching plane. When the matching plane does not pass through the isocenter, it is no longer perpendicular to the y‐axis, but has a small tilt angle. Both the couch and collimator must be rotated in order to geometrically match the tangential breast fields with the supraclavicular field. Beam matching can be achieved manually by trial and error using a 3D treatment planning software (TPS). One manually tunes the setting of the tangential fields in three view windows until they have the same divergence as the supraclavicular field. For convenience, we derived the formulae for beam matching. One can edit the formulae into a spreadsheet or implement them into Pinnacle scripts. The derivation of these formulae is given in Appendix A.

In the following sections, the collimator, couch, and gantry angles are represented by α, β, and θ, respectively. The superscripts S and T represent the supraclavicular and tangential breast fields, respectively. For example, $\theta^{T}$ is the gantry angle of a tangential breast field. The formulae are applicable to both the medial and lateral fields; therefore, medial and lateral fields are not distinguished.

#### A.1 Supraclavicular field (SCF)

The setup of the SCF is similar to the traditional three‐field monoisocentric technique, except the location of the isocenter is not within the matching plane. It can be placed inferior to the matching plane. A small (i.e., 10°–15°) gantry angle is used to avoid irradiating the trachea and the spinal cord. A block is manually drawn in the SCF to include the desired nodal regions while avoiding structures at risk, including the acromio‐clavicular joint and humerus. With the isocenter located below the block, the inferior jaw travels across the midline of the radiation field. For those machines whose jaws cannot travel across midline, one can rotate collimator 90° and use the multileaf collimator (MLC) to form the inferior border of the SCF.

For convenience, the matching plane is defined to be parallel to the x‐axis. With this assumption, the matching plane is projected into a line in the patient's sagittal view. When there is a gantry angle, the inferior plane of the SCF is not exactly parallel to the x‐axis, but has a small rotation angle, which can be corrected by a collimator rotation. The collimator angle $\alpha^{S}$ of SCF is calculated by:

(1) $$\alpha^{s}={\text{sin}}^{-1}\left(\frac{A\cdot \text{tan}\theta^{s}}{100}\right)$$

where *A*, the distance (in cm) along the y‐axis from the isocenter to the inferior plane, equals the absolute value of the inferior jaw position of the SCF field. The formula assumes that the MV source to gantry rotation axis distance is 100 cm. With collimator angle $\alpha^{S}$ and gantry angle $\theta^{S}$, the matching plane is turned parallel to the x‐axis. When the gantry angle $\theta^{S}$ is small (<10°), this collimator angle $\alpha^{S}$ is very small and can be ignored.

#### A.2 Tangential breast fields

The initial setup of the tangential breast fields is similar to that of traditional monoisocenter planning. The tangential gantry angles $\theta^{T}$ are chosen to encompass the breast or chest wall planning target volume while minimizing dose to the lung and the heart based on the patient's geometry. The medial and lateral fields are matched at the posterior border, as shown in Fig. 1(c). These fields are not exactly opposed when the isocenter is not located on the matching line. Blocks are used to shield the lung and the heart (for left‐sided treatment) in the field.

As shown in Figs. 1(a) to 1(c), in order to match the SCF with the tangential fields, the source positions (Points II and III) need to be located in the matching plane. Once the gantry angles of the tangential fields are determined, the couch angles $\beta^{T}$ of the tangential fields are calculated as:

(2) $$\beta^{T}={\text{sin}}^{-1}\left(\frac{A(\text{cos}\theta^{T}-\text{cos}\theta^{s})}{100\text{cos}\theta^{s}\text{sin}\theta^{T}\text{cos}\alpha^{s}}\right)$$

where $\theta^{T}$ is the gantry angle of the corresponding tangential field. With a couch angle $\beta^{T}$ and a gantry angle $\theta^{T}$, the MV source (Point II or III) are located in the matching plane.

The upper jaw of the tangential field should match the inferior jaw of the SCF. The collimator of the tangential fields is rotated so that the superior plane of the tangential fields coincides with the matching plane, as well as the inferior plane of the SCF. The collimator angles for the tangential fields are determined by:

(3) $$\alpha^{T}={\text{tan}}^{-1}\left(\text{tan}\beta^{T}\text{cos}\theta^{T}+\frac{A\text{sin}\theta^{T}}{100\text{cos}\beta^{T}\text{cos}\alpha^{s}\text{cos}\theta^{s}}\right),$$

where $\beta^{T}$ and $\theta^{T}$ are the couch and gantry angles of the corresponding tangential field.

When the superior plane of the tangential fields and the inferior plane of the supraclavicular field are perfectly matched, the upper jaw setting of tangential field is not exact, but very close to A. An exact geometric match does not always yield the optimal dosimetric match. Thus, the jaw setting is determined by starting the upper jaw position at A and then manually tuning this value until appropriate dose coverage is achieved in the matching plane.

**Figure 2:**
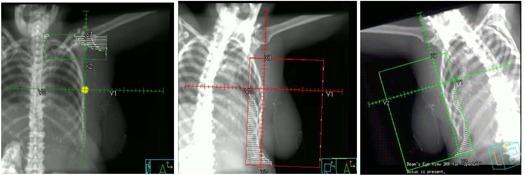
Manual multileaf collimator (MLC) blocks for the supraclavicular field (SCF) and tangential open fields. The isocenter is placed inferior to the matching plane under the SCF block.

After the three fields are geometrically matched, manual blocks are drawn in the tangential fields to block the lung and the heart. Because the tangential fields are not exactly opposed, one may need to manually edit the block contours to improve field matching at the posterior edge. A perfect geometric match at the posterior borders of the tangential fields cannot be achieved when the block has a curved edge. Figure 2 shows an example of blocks manually drawn for a breast patient. The lung and the heart were (partially) blocked. Sufficient skin flashing is given in the tangential fields in order to compensate for breast setup variation and intrafractional motion during daily treatments.

### B. Delineation of the target volumes

Commercial treatment planning software (Pinnacle; Philips Medical Systems, Fitchburg, WI) was used in this study. For convenience, we use an isodose volume to define an estimate of the breast volume. The SCF is turned off during dose calculation. After the doses from the open fields are calculated, 50% isodose lines are converted into a ROI, representing the breast volume. The breast contours are further manually edited by physicians. The involved lung, the heart, and other ROIs are also contoured manually. The skin is defined as the 3 mm outer layer of the body contoured automatically using a threshold method.

The planning target volume for evaluation (${\text{PTV}}_{\text{eval}}$) is defined as:

(4) $$PTV_{eval}=Breast-(skin\cup (heart+3mm)\cup (lung+3mm))$$

### C. Inverse planning

Again, commercial treatment planning software Pinnacle was used for inverse planning. The three‐field geometric matching technique can be used in inverse, forward, or wedge‐pair treatment planning. Conventional inverse planning generates beam apertures conformal to the target and does not provide sufficient skin flashing, which is of importance for compensating breast motion and changes. To solve this problem, we employ an open/IMRT hybrid optimization method.^(11)^ The tangential fields are duplicated prior to plan optimization. In the setting of IMRT optimization parameters, one set of tangential fields uses beam‐weight optimization during which the aperture remains the same and only MU changes. The other set of tangential fields uses direct machine parameter optimization (DMPO). Generally the intensity fluences of the breast fields are not complex, and four to five segments per beam would be sufficient for most situations. The supraclavicular field is turned off during treatment plan optimization.

IMRT optimization is an ill‐posed problem and does not yield a unique solution. Treatment plans with a higher contribution from the open fields are desired for the consideration of breast motion. The optimization starts with high initial weights for the open fields (i.e., 80%). The optimization may end up in a local minimum with higher weights for the open fields.

Beam energies and the use of bolus are chosen based on the patient's geometry and clinical characteristics. In order to deliver sufficient dose to the breast surface, lower energy (6 MV) is preferred for the open fields. The IMRT breast tangential fields may use higher energy, if necessary. If the depth that the tangential beams need to penetrate is too great, a higher energy may be used for the open fields, too. For convenience, if the corresponding open and IMRT fields have the same photon energy, they can be merged together into one beam, with the open field as the first control point (segment) of the beam. In Pinnacle TPS, there is no direct function to merge control points of different beams into one beam. A Pinnacle script has been developed for this purpose.

## III. RESULTS

**Figure 3:**
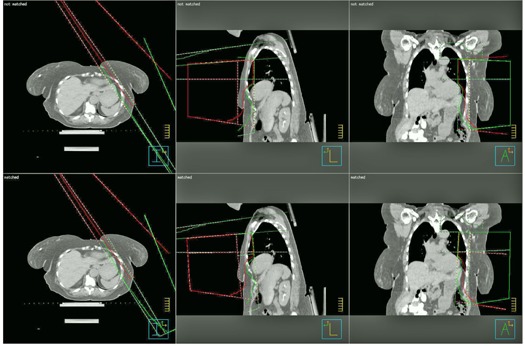
An example of isocentric three‐field matching without half‐beam blocks: (top) before field matching, (bottom) after field matching. With this method, three fields are perfectly matched geometrically.

Figure 3 shows a patient's three‐field geometry before and after geometric matching. The tangential fields were longer than 20 cm so that a conventional monoisocentric beam arrangement with half‐beam blocks could not be used. The new monoisocentric planning technique was used. After the gantry angles were determined for the SCF and tangential fields, other beam parameters were calculated using (1), (3). A Pinnacle script was developed to automatically calculate and set field parameters. The three fields were geometrically matched with the new method, as shown in Fig. 3. Table 1 lists the gantry, couch, and collimator angles before and after field matching.

**Table 1:** Field settings before and after geometry matching

	*Before Geometry Matching*			*After Geometry Matching*		
	*Gantry*	*Couch*	*Collimator*	*Gantry*	*Couch*	*Collimator*
SCF	350	0	0	350	0	359
Medial	325	0	0	325	359	358
Lateral	148	0	0	148	16	16

Lateral=tangent beam; Medial=tangent beam; SCF=supraclavicular field.

**Figure 4:**
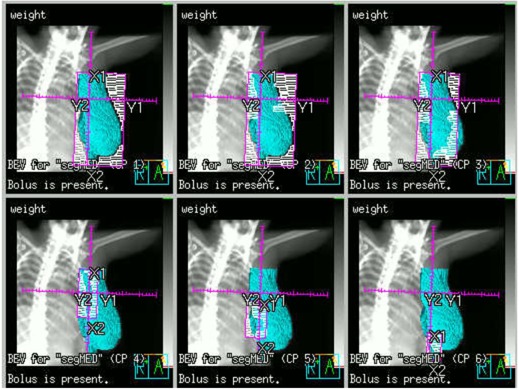
The MLC segments of one of the IMRT field after hybrid optimization. Given higher initial weights to open beams prior to optimization will result in a plan with fewer MUs for the IMRT segments, which is preferred, considering the breast motions.

Blocks were manually drawn on the open fields after the three fields were geometrically matched. ROI were delineated as described in the previous section. The breast tangential open fields were copied and blocks were removed. The second pair of tangential fields was optimized as IMRT beams with DMPO optimization, whereas the first pair was kept as open fields and optimized with beam weight. Figure 4 shows the MLC segments (control points) after optimization. The open field remains the same as shown in Fig. 2. For this particular patient, 6x and 18x beam energies were chosen for the open and tangent fields, respectively. Hence the open fields remain as separated beams. The upper jaws of the tangent fields may move and travel beyond matching plane during DMPO optimization. When SCF field is turned on, the dose in the matching plane may become too hot. If this is the case, one can manually adjust the upper jaw to A to achieve better dose distribution.

**Figure 5:**
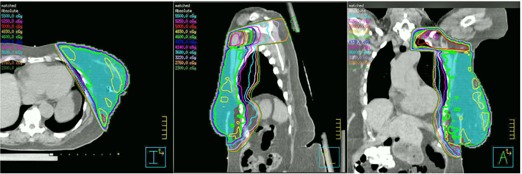
Dose distributions by the new three‐field inverse planning technique. The volume in solid cyan color is the ${\text{PTV}}_{\text{eval}}$. The new three‐field planning method with perfect geometric matching and inverse optimization can improve dose uniformity and coverage.

Figure 5 shows the dose distribution after optimization. Inverse planning has more freedom in optimization and is expected to be able to generate a plan with better dose uniformity and coverage, compared with wedge pair or forward planning methods.

**Figure 6:**
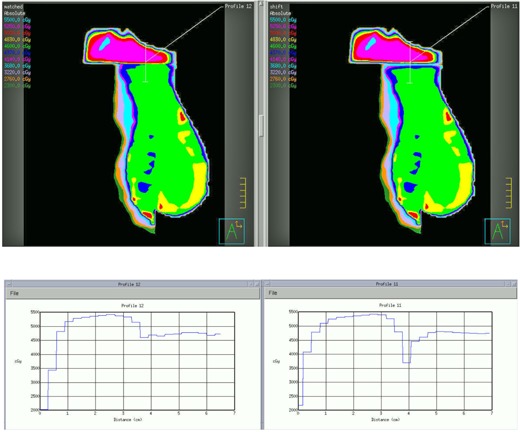
Comparisons of dose distributions without (left) and with (right) 1 mm mismatch between SCF and tangential fields. Significant underdosing (>20%) was observed in the matching plane. The dose profiles were drawn along the lines shown in the dose distributions.

Conventionally, if the tangential fields are longer than 20 cm, a dual‐isocenter beam arrangement has to be used. Delivery of such a plan requires two setups, which is not only time consuming, but also may cause large setup uncertainties. The dose gradients at the field edges are very high, thus a slight setup variation can cause significant dose difference in the matching plane. To simulate the consequence of field mismatch, we purposely shifted the SCF up by 1 mm and calculated the dose again. Figure 6 shows the dose distribution with and without the 1 mm mismatching. One can see from the dose profile that a mere 1 mm mismatch caused significant dose variation in the matching plane.

## IV. DISCUSSION

The three‐field matching technique presented in this study provides several advantages over traditional techniques. In traditional monoisocenter planning, the tangential field length is limited to within 20 cm due to half‐beam blocking; with the new technique, the full field size can be utilized in three‐field treatments so that monoisocentric three‐field setup can be used on all patients. Compared with traditional dual‐isocenter approach, the new monoisocenter method can reduce setup time and uncertainties. Although the tangential fields have small couch angles, most advanced linac machines can rotate the gantry and couch remotely. At William Beaumont Hospitals, the Elekta machines are collision‐free for all gantry angles when couch kick is within $\pm 15^{\circ }$. Therefore, unlike the dual‐isocenter approach, where therapists have to enter the room to visually inspect the patient during treatment setup, therapists do not need to go inside the treatment vault during treatment delivery.

Although this method always produces perfect geometric matching, a smaller A value is preferred in order to minimize couch rotation angles. For example, if A = 10 cmA=10 cm, the lateral couch rotation angle would be close to 20° which imposes potential collision problem. When A value is small, the medial couch angle is very small and can be ignored. This is particularly important for clinics whose patient safety policy does not allow remote couch movement at all. With only the lateral couch angle nonzero, the therapists only need enter treatment vault once in this situation.

Not only does this novel method reduce treatment time, but it also reduces uncertainties in treatment delivery. The presented monoisocentric technique reduces the dose uncertainty due to daily setup variation, as illustrated in Fig. 6. Studies showed that in clinical situations the mismatch could be much larger than the 1 mm that we simulated.^(4)^ Therefore, the underdosing in the matching plane could be significant. As the lymph node targets are often located in the matching plane, the dose coverage may be compromised even by a small setup variation.

The inverse planning technique in this study is not limited to opposed tangential fields. Despite studies showing that multiple beam inverse planning does not produce better plans than tangential fields,^12^, ^13^ in certain special situations, especially some chest wall cases, an arrangement with multiple beams may be useful. In this situation, the same beam‐matching method can be used for all fields. VMAT may also be used for chest wall planning. Because the couch angle cannot change during VMAT delivery, this field matching technique is not applicable for VMAT delivery.

Different from the traditional three‐field arrangement, in which the matching plane is always perpendicular to the y‐axis, the new beam arrangement's matching plane has a small tilt angle. This angle does not pose a significant dosimetric impact for most cases. The small tilt angle of the matching plane may make a small improvement in lung dose in some patients because the SCF passes obliquely through less lung tissue.

We assumed no initial couch angle and the matching plane is perpendicular to y‐z plane in this study. In some clinical situation, it may be useful to have a couch angle, for example to avoid patient's arm. The same formulae can still be used, but initial couch angle should be added to the couch angles calculated by Eq. (2).

## V. CONCLUSIONS

A monoisocentric three‐field matching technique has been developed and used in clinical treatments at William Beaumont Hospitals, Royal Oak, Michigan. The isocenter, shared by the supraclavicular and two tangential fields, is located inferior to the matching plane, thereby allowing utilization of full treatment field size. Perfect geometric matching is achieved by rotating the collimator and couch for the tangential fields, based on formulae described in this manuscript. This novel treatment method can shorten treatment time and reduce dose uncertainties in the three‐field matching region.

## Appendix A: Derivation of Field Matching Formula

### A. Coordinate systems

In this paper, the coordinate system is defined based on International Electrotechnical Commission (IEC) coordinate system convention. The linac is installed with couch and gantry rotation isocenters located at the origin of the world coordinate system. The gantry rotates about the y‐axis with the sign of the rotation angles defined by the right‐hand rule. A zero degree gantry angle denotes the vertical machine position with the treatment beam facing down. The couch rotates about the z‐axis with the sign of the rotation angle defined as opposite to the right‐hand rule. The MV source to axis distance is 100 cm and maximum field size is 40 cm for most commercial linacs.

### B. Collimator angles for supraclavicular field

Assume the MV source to axis distance is 100 cm. With the SCF's gantry and collimator angles set to zero, the matching plane A would be perpendicular to the y‐z plane. It intercepts z‐axis at z = 100 and y‐axis at y = A. Thereby the equation for plane A is

(A1) $$\frac{y}{A}+\frac{z}{100}=1$$

where *A* is the length of the line segment which is y‐axis intercepted by the inferior plane of the SCF. It has the same absolute value of the corresponding jaw setting. With a gantry angle $\theta^{S}$, one can rotate the collimator to make plane A independent of x.

(A2) $$\left[\begin{array}{l} x\\ y\\ z \end{array}\right]=R_{z}(-\alpha^{s})\times R_{y}(-\theta^{s})\left[\begin{array}{l} x^{\prime }\\ y^{\prime }\\ z^{\prime } \end{array}\right]=\left[\begin{array}{ccc} \text{cos}\alpha^{s} & \text{sin}\alpha^{s} & 0\\ -\text{sin}\alpha^{s} & \text{cos}\alpha^{s} & 0\\ 0 & 0 & 1 \end{array}\right]\left[\begin{array}{ccc} \text{cos}\theta^{s} & 0 & -\text{sin}\theta^{s}\\ 0 & 1 & 0\\ \text{sin}\theta^{s} & 0 & \text{cos}\theta^{s} \end{array}\right]\left[\begin{array}{l} x^{\prime }\\ y^{\prime }\\ z^{\prime } \end{array}\right]$$

where $\alpha^{S}$ is the collimator angle of the SCF, and *R* is the rotation transformation matrix. Substituting (A1), (A2), the equation for plane A after gantry and collimator rotation is

(A3) $$\left(\frac{-\text{sin}\alpha^{s}\text{cos}\theta^{s}}{A}+\frac{\text{sin}\theta^{s}}{100}\right)x+\frac{\text{cos}\alpha^{s}}{A}y+\left(\frac{\text{sin}\alpha^{s}\text{sin}\theta^{s}}{A}+\frac{\text{cos}\theta^{s}}{100}\right)z=1$$

To make the plane A parallel to the x‐axis, we have

(A4) $$\frac{-\text{sin}\alpha^{s}\text{cos}\theta^{s}}{A}+\frac{\text{sin}\theta^{s}}{100}=0$$

Therefore the collimator angle is

(A5) $$\alpha^{s}={\text{sin}}^{-1}\left(\frac{A\cdot \text{tan}\theta^{s}}{100}\right)$$

Substituting (A3), (A4), (A5), after collimator and gantry rotation, the matching plane A becomes

(A6) $$\frac{\text{cos}\alpha^{s}}{A}y+\frac{1}{100\text{cos}\theta^{s}}z=1$$

### C. Couch angles for two tangential breast fields

The superior edges of the tangential breast fields share the same plane A when they are geometrically matched with the supraclavicular field. Hence the MV source position Points II and III must be within the matching plane A. With collimator angle $\alpha^{T}$, couch angle $\beta^{T}$, and gantry angle $\theta^{T}$, the coordinate of the MV source (Points II and III) are

(A7) $$\left[\begin{array}{l} x\\ y\\ z \end{array}\right]=\left[\begin{array}{ccc} \text{cos}\beta^{T} & \text{sin}\beta^{T} & 0\\ -\text{sin}\beta^{T} & \text{cos}\beta^{T} & 0\\ 0 & 0 & 1 \end{array}\right]\left[\begin{array}{ccc} \text{cos}\theta^{T} & 0 & \text{sin}\theta^{T}\\ 0 & 1 & 0\\ -\text{sin}\theta^{T} & 0 & \text{cos}\theta^{T} \end{array}\right]\left[\begin{array}{l} 0\\ 0\\ 100 \end{array}\right]=100\left[\begin{array}{c} \text{cos}\beta^{T}\;\text{sin}\beta^{T}\\ -\text{sin}\beta^{T}\;\text{sin}\;\theta^{T}\\ \text{cos}\theta^{T} \end{array}\right]$$

Substituting (A6), (A7), one obtains

(A8) $$-\frac{100\text{sin}\theta^{T}\text{sin}\beta^{T}\text{cos}\alpha^{s}}{A}+\frac{\text{cos}\theta^{T}}{\text{cos}\theta^{s}}=1$$

Hence couch angles for the tangential breast fields are

(A9) $$\beta^{T}={\text{sin}}^{-1}\left(\frac{A\times (\text{cos}\theta^{T}-\text{cos}\theta^{S})}{100\;\text{cos}\theta^{S}\text{sin}\theta^{T}\text{cos}\alpha^{S}}\right)$$

### D. Collimator angles for two tangential breast fields

After turning Points II and III onto the matching plane A, the collimator also needs to be rotated in order to match the superior planes of the breast fields with the inferior plane of the supraclavicular field. With zero collimator and gantry angles, the collimator vector $B_{0}$ is defined as (1,0,0). When tangential fields match the SCF, the collimator vector would be parallel to the matching plane A. At gantry angle $9^{T}$, couch angle $\beta^{T}$, and collimator angle $\alpha^{T}$, the collimator vector $B_{0}$ becomes

(A10) $$\begin{array}{c} \vec{B}=\left[\begin{array}{ccc} \text{cos}\beta^{T} & \text{sin}\beta^{T} & 0\\ -\text{sin}\beta^{T} & \text{cos}\beta^{T} & 0\\ 0 & 0 & 1 \end{array}\right]\left[\begin{array}{ccc} \text{cos}\theta^{T} & 0 & \text{sin}\theta^{T}\\ 0 & 1 & 0\\ -\text{sin}\theta^{T} & 0 & \text{cos}\theta^{T} \end{array}\right]\left[\begin{array}{ccc} \text{cos}\alpha^{T} & -\text{sin}\alpha^{T} & 0\\ \text{sin}a^{T} & \text{cos}\alpha^{T} & 0\\ 0 & 0 & 1 \end{array}\right]\cdot {\vec{B}}_{0}\\ =\left[\begin{array}{c} \text{cos}\beta^{T}\text{cos}\theta^{T}\text{cos}\alpha^{T}+\text{sin}\beta^{T}\text{sin}\alpha^{T}\\ -\text{sin}\beta^{T}\text{cos}\theta^{T}\text{cos}\alpha^{T}+\text{cos}\beta^{T}\text{sin}\alpha^{T}\\ -\text{sin}\theta^{T}\text{cos}\alpha^{T} \end{array}\right] \end{array}$$

When the tangential fields match the supraclavicular field, vector ${\vec{B}}_{0}$ is parallel to plane A, which is defined by Eq. (A6). Hence we have

(A11) $$\frac{-\text{sin}\beta^{T}\text{cos}\theta^{T}\text{cos}\alpha^{T}+\text{cos}\beta^{T}\text{sin}\alpha^{T}}{-\text{sin}\theta^{T}\text{cos}\alpha^{T}}=\frac{-A}{100\;\text{cos}\theta_{y}^{S}\;\text{cos}\alpha^{S}}$$

The collimator angles for tangential fields are thereby obtained by

(A12) $$\alpha^{T}={\text{tan}}^{-1}\left(\text{tan}\beta^{T}\text{cos}\theta^{T}+\frac{A\text{sin}\theta^{T}}{100\text{cos}\beta^{T}\text{cos}\alpha^{S}\text{cos}\theta^{S}}\right)$$
